# The Gut Microbiota of *Drosophila melanogaster*: A Model for Host–Microbe Interactions in Metabolism, Immunity, Behavior, and Disease

**DOI:** 10.3390/microorganisms13112515

**Published:** 2025-10-31

**Authors:** Kyu Hong Cho, Song Ok Kang

**Affiliations:** 1 Department of Biology, Indiana State University, 600 Chestnut St. S224, Terre Haute, IN 47809, USA; 2 Department of Applied Medicine and Rehabilitation, Indiana State University, Terre Haute, IN 47809, USA

**Keywords:** *Drosophila melanogaster*, host–microbe interactions, gut microbiota, *Lactobacillus*, *Acetobacter*, peptidoglycan, acetate, cyclic di-nucleotides, uracil, TOR/insulin signaling, DUOX pathway, STING signaling

## Abstract

The gut microbiota of *Drosophila melanogaster* offers a simplified yet powerful system to study conserved mechanisms of host–microbe interactions. Unlike the highly complex mammalian gut microbiota, which includes hundreds of species, the fly gut harbors a small and defined community dominated by *Lactobacillus* and *Acetobacter*. Despite its low diversity, this microbiota exerts profound effects on host physiology. Commensal bacteria modulate nutrient acquisition, regulate insulin/TOR signaling, and buffer dietary imbalances to support metabolic homeostasis and growth. They also influence neural and behavioral traits, including feeding preferences, mating, and aggression, through microbial metabolites and interactions with host signaling pathways. At the immune level, microbial molecules such as peptidoglycan, acetate, uracil, and cyclic dinucleotides activate conserved pathways including Imd, Toll, DUOX, and STING, balancing antimicrobial defense with tolerance to commensals. Dysbiosis disrupts this equilibrium, accelerating aging, impairing tissue repair, and contributing to tumorigenesis. Research in *Drosophila* demonstrates how a low-diversity microbiota can shape systemic host biology, offering mechanistic insights relevant to human health and disease.

## 1. Introduction

The human gut harbors hundreds of different microbial species spanning many different phyla, forming a highly complex and dynamic ecosystem. This complexity poses substantial challenges for scientists attempting to decipher causal relationships between specific microbes and host health or disease. Contrasting sharply with the complexity of the human microbiota, the gut microbiota of *Drosophila melanogaster*, commonly known as the fruit fly, is far less diverse than mammals’ gut microbiota, typically comprising fewer than 20 culturable species. These bacteria mainly belong to two major groups: Proteobacteria, which include species like *Acetobacter* and *Gluconobacter*, and Firmicutes, mainly represented by *Lactobacillus* and *Enterococcus*. This relatively limited and well-defined microbial community makes *Drosophila* an extraordinarily useful model organism for studying the complex interactions between hosts and their gut microbes [1,2]. Recent metagenomic analyses have further revealed that while the core gut microbiota is dominated by a few genera, transient microbes from the environment can temporarily colonize the gut, influencing host responses [3,4].

Despite its simplicity, the *Drosophila* gut microbiota shares many core functional characteristics with mammalian gut microbiota. Both systems show that microbial communities are highly responsive to dietary changes, with shifts in microbial composition reflecting the types of food consumed [2]. Furthermore, the *Drosophila* gut microbes influence host energy balance by affecting nutrient absorption and metabolism, and they are essential for proper immune system development and function, priming innate immune defenses and modulating susceptibility to infections [5,6,7]. Recent studies have demonstrated that dysbiosis can accelerate aging and neurodegenerative processes, mirroring human conditions such as Parkinson’s disease [8,9,10]. These conserved functional roles make *Drosophila* an invaluable model for investigating how dysbiosis in the gut microbiota may contribute to a variety of diseases, including infection [11,12], aging [13,14], cancers [15], inflammatory bowel disease (IBD) [16,17], and metabolic disorders [18].

In *Drosophila*, researchers can manipulate the gut microbiota, often using germ-free (axenic) or gnotobiotic flies, to directly test how particular bacteria influence host physiological processes, immune responses, and development [1,19]. Advances in gnotobiotic techniques have enabled mono-association studies with single bacterial strains, revealing strain-specific effects on host lifespan and metabolism [20,21,22,23].

While *Drosophila* cannot fully replicate the complexity of the human microbiota, its simplified and genetically tractable system reveals fundamental, evolutionarily conserved mechanisms governing host–microbe interactions. Insights gained from this model organism provide a crucial foundation for further validation in mammalian systems and may eventually lead to clinical innovations targeting gut microbiota-related diseases.

## 2. Drosophila Gut

**Figure 1:**
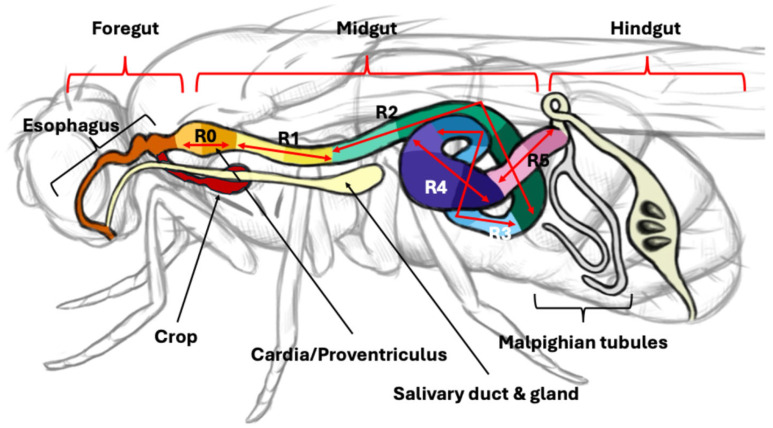
Schematic diagram of the adult *Drosophila melanogaster* digestive tract. The gut is anatomically and functionally divided into three major regions: the foregut, midgut, and hindgut. The foregut includes the esophagus and crop, functioning in food storage and initial mechanical digestion. The midgut, the primary site for enzymatic digestion and nutrient absorption, is further subdivided into functional regions (R0–R5) based on differences in physiology, cell types, and microbial composition. Arrows indicate each functional regions (R0–R5), and different colors show subregions. The hindgut is involved in water reabsorption and waste excretion. The illustration of *D. melanogaster* and its gut anatomy was partly sourced from figshare.com, which provides unlabeled figures [38].

**Figure 2:**
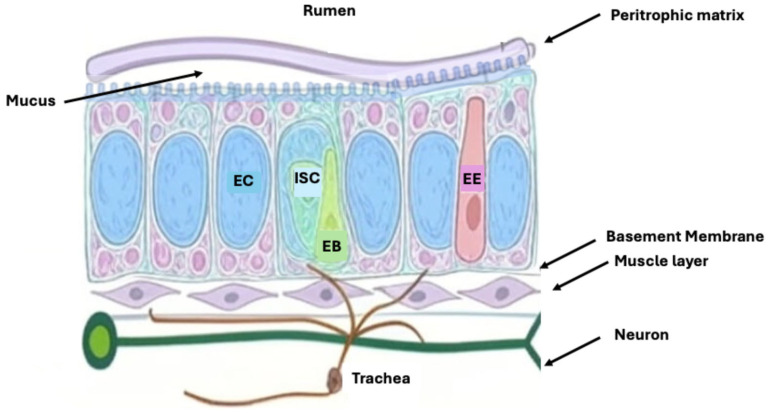
Cellular organization of the adult *Drosophila* midgut epithelium. The diagram shows a cross-sectional view of the midgut epithelium, highlighting its structural and cellular components. The peritrophic matrix lines the gut lumen and acts as a semi-permeable barrier between ingested material and epithelial cells. The epithelium is primarily composed of enterocytes (ECs), which are large absorptive cells, and enteroendocrine cells (EEs), which secrete hormones. Intestinal homeostasis is maintained by intestinal stem cells (ISCs), which divide to give rise to enteroblasts (EBs), transient progenitors that differentiate into ECs or EEs. Supporting structures include the basement membrane, muscle layer, neurons, and trachea, which provide structural integrity, motility, innervation, and oxygen supply, respectively. This arrangement allows for dynamic maintenance and regeneration of the gut epithelium in response to internal and external stimuli.

The digestive tract of *D. melanogaster* is a tubular organ composed of a single epithelial cell layer. This organ is divided into three main sections, each with a specialized role, namely, the foregut, midgut, and hindgut (Figure 1) [24,25]. The entire gut lining is protected by the peritrophic membrane (PM), a semi-permeable, chitinous barrier that separates the epithelial cells from the gut lumen contents (Figure 2). This membrane acts as a physical barrier, protecting the epithelium from mechanical damage and preventing harmful microbes and toxins from directly contacting the tissue [26].

This initial section includes the mouth, esophagus, and crop. It functions primarily as a storage organ and a site for the beginning of digestion, where ingested food is stored and initial breakdown occurs. The crop can hold a large volume of food, allowing the fly to regulate feeding and digestion efficiently. The foregut, particularly the crop and proventriculus at the junction between the foregut and midgut, serves as a specialized niche for stable microbial colonization. Unlike other regions of the gut where microbes are typically transient, the foregut supports long-term association with specific commensal bacteria, most notably strains of *Lactobacillus* and *Acetobacter* [27]. These bacteria selectively colonize crypt-like structures in the proventriculus, where the host secretes mucus-like substances and forms extracellular matrices. Importantly, primary colonizers can shape the niche environment, facilitating the integration of additional microbial species [27].

Originating from endodermal tissue, the midgut is the central hub for digestion and nutrient absorption. It is where enzymes such as carbohydrases, proteases, and lipases break down complex nutrients into simpler forms that can be absorbed into the bloodstream. The midgut is structurally complex, subdivided into six primary regions labeled R0 through R5, each with distinct functions and gene expression profiles, which are further divided into more specific subregions [28,29]. At least ten subregions have been identified, each characterized by unique cell morphology, specialized digestive and absorptive functions, and regional gene expression profiles (Figure 2) [29]. Single-cell RNA sequencing recently uncovered heterogeneous cell populations within these regions, including distinct enterocyte subtypes [30]. Different subregions harbor varying densities and compositions of bacterial communities, reflecting the selective pressures imposed by regional immune activity, local pH, and nutrient gradients. The anterior midgut hosts the highest burden of commensal microbes and exhibits the highest expression of antimicrobial peptides, serving as the primary site for microbial colonization [31]. The middle midgut contains copper cells that acidify the lumen, lowering the pH to less than 4, which acts as a barrier to microbial passage, thus restricting microbial presence in this compartment [32]. The posterior midgut is relatively microbe-free, except during overwhelming infection, and is primarily involved in nutrient absorption [32].

Derived from ectodermal tissue, the hindgut plays a crucial role in water reabsorption, ion regulation, and waste excretion. It works in concert with the Malpighian tubules, functionally similar to vertebrate kidneys, to maintain water and electrolyte balance, especially in response to environmental changes [24]. The hindgut’s functions resemble those of the human colon, especially in processing waste and regulating water reabsorption. The hindgut maintains a slightly acidic pH, which helps in controlling microbial populations and detoxifying ingested substances. Most ingested bacteria do not survive passage through the acidic zone of the midgut, indicating that the hindgut is not a major site for microbial colonization.

When designing a gut microbiota experiment in *Drosophila melanogaster*, the first consideration is which part of the gut to sample. Each gut compartment has distinct physiology and bacterial density, so the choice of tissue should align with the research question. Whole-gut sampling (i.e., homogenizing the entire gut or whole fly) is commonly used when the goal is to capture the complete microbial community or to compare overall bacterial load across conditions [3,33,34]. In contrast, when the focus is on host-mediated selection, metabolism, or immunity, researchers often sample the midgut, particularly the anterior and middle regions [33,35,36,37]. The midgut is the primary site of digestion and nutrient absorption in *Drosophila*, and it also serves as the frontline of host defense against microbes. Epithelial cells in this region produce antimicrobial peptides and reactive oxygen species, creating a dynamic environment that shapes microbial composition. As a result, the midgut often reflects the balance between digestion, immunity, and colonization. For this reason, many studies target the midgut to investigate how host digestive physiology influences the gut microbiota or how specific bacterial species modulate gut immunity.

## 3. Microorganisms in the *Drosophila* Gut Microbiota

Unlike mammals, which often maintain a stable, long-term association with their gut microbes, *Drosophila* primarily acquires its microbiota from the environment, mainly through feeding on fermenting fruit and decaying organic matter. This results in a highly dynamic and transient microbial community that is constantly renewed with each generation, with microbes being shed with feces and replaced by new microbes from the environment [39]. *Drosophila* gut microbiota is maintained by selective colonization, diet, and immune activity.

Despite exposure to many microbes in the environment, only a select few, most notably *Lactobacillus* and *Acetobacter*, successfully colonize the *Drosophila* gut [6,40]. These microbes are resistant to environmental stresses and possess specific adaptations that enable them to adhere to the gut epithelium, allowing for functional colonization [41].

Diet plays a crucial role in shaping the gut microbiota. Dietary changes can rapidly and profoundly alter gut microbial communities, often with significant physiological consequences. Diets rich in fermentable sugars support the proliferation of *Lactobacillus* and *Acetobacter* species, which in turn help enhance nutrient absorption, stimulate growth, and regulate host metabolic pathways [42,43,44]. High-fat diet (HFD) consumption in *Drosophila* alters the composition of gut microbiota [45]. HFD markedly increases the levels of *Acetobacter malorum* in the gut, and this change leads to the activation of the proinflammatory Immune deficiency (Imd)/NF-κB immune signaling pathway in the head. Introducing *A. malorum* to *Drosophila* alone is sufficient to induce the inflammatory responses in the head. *A. malorum* produces elevated levels of peptidoglycan (PGN), a recognized microbial molecular signal, which enters systemic circulation and triggers the inflammation remotely.

The fly’s innate immune system tightly controls microbial populations in the gut through pathways like Imd, DUOX, and *Drosophila* STING (dSTING). These pathways detect bacterial molecules such as PGN, uracil, and cyclic dinucleotides (CDNs), respectively, and regulate the production of anti-microbial peptides (AMPs) and reactive oxygen species (ROSs) to keep microbial populations in check. This aspect will be more described in Section 4.

The microbial community within the *Drosophila* gut is predominantly bacterial and is characterized by its low diversity. The most abundant groups are shown below.

### 3.1. Lactobacillaceae

*Lactobacillus* species contribute to carbohydrate fermentation, help maintain gut barrier integrity, and stimulate immune responses. They are often considered probiotic microbes that support host health and resistance to infections. Symbiotic *Lactobacillus* species, including *Lactobacillus rhamnosus* GG, *Lactobacillus plantarum*, and *Lactobacillus salivarius*, stimulate Nox1-dependent ROS production, driving intestinal stem cell proliferation in both mice and flies [46]. The most prominent species in the gut of *D. melanogaster* are *L. plantarum*, *Lactobacillus brevis*, and *Lactobacillus fructivorans* [47].

***L. plantarum*** mono-associated *Drosophila* demonstrated that the presence of *L. plantarum* is sufficient to accelerate larval growth under poor nutritional conditions [41]. The bacterium also benefits adults: association with *L. plantarum* yields earlier emergence of fit, fertile adults on poor diets, consistent with the growth-promotion phenotype [48]. Mechanistically, *L. plantarum* alters levels of host nutrients or nutrient-related signals, and host Target of Rapamycin (TOR)/insulin signaling is required for the microbial growth-promoting effect [41]. *L. plantarum* upregulates intestinal peptidase expression and proteolytic activity, boosting host amino acid levels; this effect is partly Imd/Relish-dependent and can be antagonized by pathogen challenge [49]. Transposon screening and targeted mutants of *L. plantarum* identified *pbpX2-dlt* (D-alanylation of teichoic acids) as required for *L. plantarum*-mediated growth promotion; D-Ala-LTAs act as direct cues sensed by the host to induce intestinal peptidases and juvenile growth during chronic undernutrition [50]. A recent study demonstrated that *L. plantarum* can rescue growth on amino acid-imbalanced diets even when it cannot synthesize the limiting amino acid; instead, bacterial ribosomal and transfer RNA (r/tRNA)-encoded cues packaged in vesicles activate the general control nonderepressible 2 kinase (GCN2) in enterocytes to remodel gut physiology and support anabolic growth [51]. In contrast, a study has reported a negative effect of *L. plantarum*: adult *D. melanogaster* intestinal balance is disrupted by mono-colonization with *L. plantarum* [20].

***L. brevis*** is a common member of both laboratory and wild *D. melanogaster* gut microbiota. Mono-association of axenic flies with *L. brevis* restores normal walking speed and daily activity; this effect was traced to the bacterial enzyme xylose isomerase, which modulates host sugar metabolism and signals through octopaminergic neurons to affect locomotion [21]. Beyond these beneficial traits, *L. brevis* has been shown to play a dual role in the fly gut. On the one hand, it can modestly support larval development and host physiology under nutrient-limited conditions [44]. On the other hand, specific strains act as pathobionts: for example, *L. brevis* strain EW continuously secretes uracil, which chronically activates DUOX-dependent ROS production in the intestinal epithelium. While acute ROS production is protective, chronic activation causes epithelial cell death, inflammation, and reduced host lifespan [52]. This demonstrates that *L. brevis* can function either as a commensal partner or as a pathobiont depending on the strain and host context. In addition, interactions with other microbes strongly influence the role of *L. brevis*. For example, co-colonization with *Acetobacter tropicalis* produced a stronger reduction in host triacylglyceride (TAG) levels and body weight than either species alone [43]. This shows that *L. brevis* not only exerts direct effects on host physiology but also contributes to synergistic metabolic outcomes through interspecies interactions. More recently, *L. brevis* was shown to promote tumor growth in Notch-deficient intestines of *D. melanogaster*. In this model, *L. brevis*, but not *L. plantarum*, enhanced tumor expansion through bacterial cell wall components rather than metabolic activity [53]. Tumors further altered the gut environment, weakening immune defenses and allowing increased colonization by *L. brevis*. This, in turn, fueled tumor progression, creating a self-reinforcing cycle between microbial growth and disease. At the cellular level, *L. brevis* disrupted integrin signaling in intestinal progenitor cells, impairing adhesion and polarity and shifting stem cell divisions toward symmetric self-renewal. The expansion of the stem cell pool at the expense of differentiation contributed directly to tissue overgrowth and tumorigenesis.

***L. fructivorans*** is a consistent and abundant member of the *D. melanogaster* gut microbiota, particularly dominant in early-instar larvae and young adults, where it can represent more than half of the microbial community. As flies age, its relative abundance declines, with *Acetobacter* species, particularly *A. pomorum*, becoming more prevalent [47]. Unlike *L. plantarum* and *L. brevis*, which accelerate larval development and shorten the time to pupation and eclosion, *L. fructivorans* has little influence on developmental rate, with its effects closely resembling those of axenic flies [43]. Metabolically, *L. fructivorans* contributes less to host lipid regulation and metabolic homeostasis than *L. plantarum* and *L. brevis* [43]. An interesting feature of *L. fructivorans* is its antagonistic relationship with *Acetobacter*, another key gut symbiont. Whereas *L. brevis* supports mutual growth with *Acetobacter* and *L. plantarum* mildly promotes its colonization, the presence of *Acetobacter* sharply suppresses *L. fructivorans* abundance, reducing its levels by more than thirtyfold [43]. Ecologically, *L. fructivorans* and *L. brevis* are transmitted through egg surfaces and maintained within both flies and their food environment. However, they compete poorly in food alone, often being outcompeted by *Acetobacter*. Their persistence is therefore favored when flies are present in higher density, suggesting that host activities modify the environment in ways that promote their stability [47].

### 3.2. Acetobacteraceae

In wild *D. melanogaster* populations, Acetobacteraceae (the family including *Acetobacter* and *Gluconobacter*) are among the predominant bacterial taxa, frequently alongside Lactobacillaceae and Enterobacteriaceae [54]. They ferment dietary sugars into acetic acid, which not only acidifies the gut environment, but also helping to inhibit harmful pathogens. They also influence host physiology and engage in metabolic cross-talk with co-resident microbes such as *Lactobacillus*. Their effects on the host are highly dependent on both the strain and the ecological context. Some strains can reproduce many of the developmental and metabolic benefits, whereas others display weaker activity or distinct functional outcomes [44].

***Acetobacter pomorum*** is a dominant member of the *Drosophila* gut microbiota. Through a combination of metabolite production, immune and endocrine modulation, and synergistic interactions with other microbes, *A. pomorum* acts as a central driver of juvenile growth in *Drosophila*. It ensures that developing larvae can adapt to nutrient fluctuations and achieve proper developmental timing, establishing it as a keystone member of the fly gut microbiota. Its growth-promoting effect depends on pyrroloquinoline quinone–dependent alcohol dehydrogenase (PQQ-ADH), which generates acetic acid that activates host insulin/insulin-like growth factor signaling, insulin/IGF signaling (IIS) [44]. Through this pathway, *A. pomorum* supports normal larval growth, metabolic balance, and intestinal stem cell renewal. Mutant strains lacking PQQ-ADH fail to provide these benefits, but dietary acetic acid or enhanced host IIS can restore development. *A. pomorum* also regulates host growth through Imp-L2, an insulin-binding protein that usually inhibits insulin signaling [55]. Colonization with *A. pomorum* suppresses Imp-L2 expression via ecdysone hormone signaling, further enhancing insulin pathway activity and promoting faster development. Beyond signaling, *A. pomorum* provides thiamine (vitamin B1), which is required for proper larval growth. In germ-free flies raised on thiamine-deficient diets, development stalls, but colonization with *A. pomorum* restores growth, highlighting its importance as a nutritional symbiont [55]. *A. pomorum* also engages in cooperative interactions with other gut microbes, notably *L. plantarum*. In this partnership, *A. pomorum* supplies amino acids and vitamins, while *L. plantarum* contributes lactate, which stimulates *A. pomorum*’s amino acid production. Together, they enhance larval growth more effectively than either species alone, particularly under poor nutritional conditions. *A. pomorum* also acts as a metabolic stabilizer, protecting the host from dietary perturbations that would otherwise disrupt lipid balance [56]. Minor differences in fly diets, such as yeast source or preservative type, were shown to strongly influence host lipid metabolism and the effects of the microbiota. Triacylglyceride (TAG) levels reflected not just microbial status, but a three-way interaction among diet composition, preservatives, and bacterial colonization. Preservatives alone altered TAG in germ-free flies, and in some cases even reversed the expected microbial effects. Among individual species, *A. pomorum* played a unique protective role: despite its growth being suppressed by preservatives, it consistently buffered against preservative-induced lipid accumulation, stabilizing host metabolic balance under dietary stress.

***Acetobacter persici*** has emerged as a bacterial species with remarkable influence over the fly’s physiology and lifespan. Flies associated with *A. persici* displays distinctive changes in purine metabolism, particularly the accumulation of allantoin, a metabolite linked to oxidative stress and tissue aging [57]. This accumulation activates the Imd pathway. As the result, the fly’s immune system remains in a low-level activated state, creating a form of metabolic inflammation that gradually accelerated aging. Exposure to *A. persici* leads to a significant reduction in lifespan and marked intestinal stem cell overproliferation, a hallmark of gut aging [58]. Even heat-killed cells of *A. persici* reproduce the same effects, the key trigger for which is peptidoglycan. These molecules are sensed by the fly’s PGRP-LC receptor, which activates the Imd pathway in the anterior region of the midgut. This signaling cascade induces the production of antimicrobial peptides, such as Diptericin A, leading to a chronic inflammatory state in the gut epithelium. Over time, this continuous immune activation disrupts gut homeostasis, stimulates excessive stem cell proliferation, and causes premature deterioration of intestinal tissue. In contrast, *L. plantarum* activates a different immune receptor (PGRP-LE) and promotes regulatory responses rather than inflammation. Despite its detrimental influence on longevity, *A. persici* provides certain short-term benefits. Flies exposed to the bacterium shows greater resistance to oxidative stress and increased survival after oral infection with *Pseudomonas entomophila*. This dual nature highlights a biological trade-off: *A. persici* enhances the host’s immediate defense and stress tolerance but undermines long-term vitality.

***Gluconobacter*** play a multifaceted role in shaping host physiology and health in *Drosophila*. Under normal conditions, they are minor constituents of the gut community, but certain stresses can drive their expansion. For example, tissue necrosis in the wings leads to an overgrowth of *Gluconobacter* in the intestine, which in turn triggers excessive activation of the Imd immune pathway, disrupts host metabolic balance, and reduces lifespan [59]. Experimental introduction of an isolated *Gluconobacter* strain confirmed that its presence alone can intensify these immune responses in susceptible flies [59]. Age-related changes in the gut also favor the proliferation of *Gluconobacter*, along with other opportunistic microbes such as *Providencia* and Enterobacteriaceae [60].

### 3.3. Enterobacteriaceae

Members of the family Enterobacteriaceae are frequently detected in the gut microbiota of *D. melanogaster*, especially in natural populations where microbial diversity is broader than in the laboratory. Genera such as *Providencia*, *Serratia*, *Erwinia*, and *Pantoea* are often present, sometimes behaving as opportunistic pathogens but nonetheless constituting part of the fly’s commensal community [39]. One of the most prominent roles of Enterobacteriaceae emerges during aging, when an expansion of Gammaproteobacteria, including many Enterobacteriaceae, drives gut dysbiosis. This microbial shift precedes intestinal barrier breakdown, stimulates systemic immune activity, and ultimately shortens host lifespan [13]. Ecological comparisons across multiple *Drosophila* species indicate that Enterobacteriaceae are common across diets and habitats, but their abundance is highly variable, being far greater in wild populations than in laboratory strains that harbor a more restricted set of taxa [61].

***Providencia*** species are natural members of the *D. melanogaster* gut microbiota and vary widely in their interaction with the host [62]. Comparative infection studies show that *P. sneebia* and *P. alcalifaciens* are highly virulent, causing almost complete mortality, while *P. rettgeri* and *P. burhodogranariea* are less pathogenic, producing moderate lethality [62,63]. Genomic comparisons reveal a shared core genome alongside species-specific genes, including adhesion factors and type III secretion systems (T3SS), which contribute to host colonization and virulence [63]. In particular, *P. alcalifaciens* carries two distinct T3SS systems that enable intracellular proliferation and rapid host killing, illustrating diversification from commensal to pathogen [64]. The balance between host and *Providencia* is strongly influenced by environment and diet. A high-sugar diet increases susceptibility to *P. rettgeri* by impairing antimicrobial peptide (AMP) protein production, leading to greater bacterial proliferation and mortality [65], while deficiency in the intestinal biotin transporter favors overgrowth of *P. sneebia*, causing oxidative stress and epithelial damage [66]. *Providencia* has also shaped the evolution of *Drosophila* immunity. Natural polymorphisms in the AMP Diptericin A affect survival after *P. rettgeri* infection, reflecting balancing selection [67], whereas Diptericin B specializes in controlling *Acetobacter*, showing microbe-specific adaptation [68]. Moreover, sublethal *P. rettgeri* exposure induces Imd-dependent innate immune priming requiring Diptericin B, improving survival on reinfection and reducing pathogen transmission [69].

***Serratia marcescens*** is an opportunistic pathogen of *D. melanogaster* that infects both through septic injury and oral routes. Septic infection rapidly kills flies within a day, as the bacterium resists systemic immune defenses, while oral infection allows it to cross the gut epithelium and establish lethal infections over several days [70]. Gut barrier structures play a critical role in limiting its pathogenicity: flies with defects in the peritrophic matrix show markedly increased susceptibility, demonstrating the PM’s function as a frontline physical and immune barrier [26]. Beyond acute infection, *S. marcescens* can persist chronically in flies, maintaining antimicrobial peptide expression and providing long-term protection against secondary infections. Such chronic infections enhance both resistance and tolerance, even to unrelated pathogens like *P. rettgeri*, revealing a form of cross-protection [71,72]. The outcome of infection is also shaped by host metabolism. High-sugar diets increase susceptibility by promoting faster bacterial growth in the gut and heightening mortality [65].

### 3.4. Yeasts

**Yeasts** are important components of the *D. melanogaster* gut microbiota, providing key nutritional benefits that shape host development and survival. Species-specific effects are shown: *Hanseniaspora uvarum* accelerates larval development, whereas *Metschnikowia pulcherrima* increases mortality [73]. Yeast-derived nutrients also affect larval growth, body weight, and developmental timing, with larvae preferring species that enhance survival [74]. Beyond nutrition, yeasts impact behavior and reproduction. Exposure to different yeast species during development alters adult fecundity, mating success, and cuticular hydrocarbon profiles, thereby influencing sexual signaling and mate choice [73]. In ecological settings, *Drosophila* are drawn to yeast volatiles and metabolites, which guide feeding and oviposition preferences [74]. Yeasts also interact with host immunity and gut environment. Persistence varies by species: some resist reactive oxygen species (ROS) and survive longer in the gut, while the host DUOX pathway restricts overgrowth and prevents pathology [74]. Additionally, yeast symbionts contribute to environmental adaptation, as shown by *Starmerella bacillaris*, which enhances reproductive efficiency on salt-rich diets [75]. Finally, yeast transmission across metamorphosis requires bacterial partners, with *S. cerevisiae* carried into adulthood only when co-associated with Enterobacteriaceae [76].

### 3.5. Viruses

**Viruses** in the *D. melanogaster* gut range from disruptive pathogens to persistent, asymptomatic residents. *Drosophila* A virus (DAV) chronically infects the midgut, driving intestinal stem cell hyperproliferation, dysplasia, barrier breakdown, and reduced lifespan via Sting-Relish, EGFR, and JNK (c-Jun N-terminal Kinase) signaling, linking viral infection to premature gut aging [77]. In contrast, Nora virus establishes persistent fecal-oral infections confined to 5–15% of midgut cells, with no obvious pathology, yet induces progressive immune gene activation and alters bacterial community diversity [78,79,80,81]. Importantly, interactions with commensal bacteria modulate antiviral defense: *A. pomorum* primes NF-κB—dependent Pvf2 expression that synergizes with viral signals to activate ERK in enterocytes, restricting replication of enteric viruses such as *Drosophila* C virus [82].

## 4. Microbial Molecules That Influence *Drosophila* Gut Immunity

The *Drosophila* gut serves as the first line of defense against a wide array of ingested microorganisms, including bacteria, fungi, viruses, and parasites, many of which are encountered naturally through feeding on decaying fruit, plants, or other organic matter. To protect against potential infections, the fly’s gut has evolved multiple intricate layers of immune defenses that recognize microbial substances to regulate immune activity [26].

### 4.1. Acetate Activating the Imd Signaling Pathway

Acetate produced by commensal bacteria, especially *Acetobacter* species such as *A. pomorum*, acts as an important regulator of *Drosophila* gut immunity. Rather than functioning simply as a byproduct of bacterial metabolism, acetate actively stimulates the Imd signaling pathway in the anterior midgut, specifically within enteroendocrine cells (EECs) [83]. This immune activation leads to the production of antimicrobial peptides, including Diptericin and Cecropin [83]. For acetate to exert its effect, it must be transported into EECs through the monocarboxylate carrier Tarag, where it is converted into acetyl-CoA. This metabolite serves as a substrate for the Tip60 histone acetyltransferase complex, promoting histone acetylation and chromatin remodeling that enhance transcription of immune-related genes, including PGRP-LC. In this way, acetate reinforces its own signaling by boosting receptor levels and sustaining basal immune activity, even in the absence of infection [84]. Beyond immune activation, this acetate-induced Imd signaling in EECs also increases the production of the endocrine peptide tachykinin. Tachykinin then acts on enterocytes to repress intestinal lipid synthesis, thereby preventing lipid overaccumulation in the gut [84]. This process links microbial metabolism to the host’s energy regulation and lipid balance.

Acetic acid also maintains a metabolic environment in which immune signaling is restrained toward beneficial bacteria. When microbial acetate production is disrupted, either by removing *A. pomorum* or by generating germ-free (axenic) flies, the host exhibits impaired IIS activity, increased intestinal lipid accumulation, and aberrant immune gene expression. These effects can be reversed by acetate supplementation, demonstrating that acetate directly mediates host tolerance by preserving homeostatic signaling [44]. Hang et al. explored how this same metabolic axis is disrupted during infection [85]. The authors identified a *Vibrio cholerae* two-component regulatory system, CrbRS, which activates acetyl-CoA synthase 1 (ACS-1) and drives the bacterial acetate switch, a shift from acetate excretion to consumption. This pathogen-mediated depletion of intestinal acetate causes deactivation of host IIS, enterocyte lipid accumulation, and intestinal steatosis, leading to host lethality. Notably, uninfected germ-free flies that lack commensal-derived acetate exhibit the same IIS suppression and lipid pathology, both of which are alleviated by dietary acetate supplementation. Together, these studies show that commensal-derived acetate acts as a metabolic signal that maintains insulin signaling, epithelial energy balance, and immune quiescence, thereby allowing *D. melanogaster* to coexist peacefully with its gut microbiota. Conversely, depletion or hijacking of acetate metabolism, whether by loss of commensals or by pathogens that scavenge acetate, disrupts this tolerance, leading to inflammation, lipid dysregulation, and host pathology. In essence, microbial acetate is both a metabolic stabilizer and a tolerance signal, preserving mutualism by aligning host energy metabolism with controlled immune responsiveness.

### 4.2. Peptidoglycan (PGN) Regulating Pattern Recognition and Signaling Pathways

Bacterial cell wall components, particularly peptidoglycan (PGN), serve as critical signals for the *Drosophila* immune system. When microbes breach the physical barriers, the fly’s immune system detects them through specialized proteins called Pattern Recognition Receptors (PRRs), particularly the Peptidoglycan Recognition Proteins (PGRPs), which detect different types of PGN depending on their chemical structure (Table 1). PGRPs are defined by the presence of a 160 amino acid PGRP domain and are conserved from insects to mammals [86]. *Drosophila* has 13 PGRP genes encoding 19 known proteins [87]. These receptors recognize peptidoglycan (PGN), a key component of bacterial cell walls. For example, PGRP-LC (membrane-bound) and PGRP-LE (cytoplasmic) are the main sensors for diaminopimelic acid (DAP)-type PGN, which is characteristic of Gram-negative bacteria like *Gluconobacter* and *Enterobacter* [88,89,90,91,92,93]. Activation of these receptors triggers the Imd pathway, leading to the activation of the NF-κB transcription factor Relish, which then promotes the production of antimicrobial peptides (AMPs). These peptides help eliminate bacteria that pose a threat to the host. PGRP-SA recognizes Lys-type PGN found mainly in Gram-positive bacteria, activating the Toll pathway, a pathway similar to mammalian TLR signaling, resulting in the production of AMPs and other immune molecules [94,95]. Some *Drosophila* PGRPs have enzymatic function. For example, PGRP-LB, PGRP-SC, and PGRP-SB remove peptides from the glycan chains of peptidoglycan [91,96,97]. These enzymatic PGRPs either modulate the immune response by scavenging peptidoglycan or act as bactericidal molecules [91,97,98].

Microbial PGN serves also as a tolerance modulator, allowing the fly to balance antimicrobial defense with symbiotic coexistence. PGN derived from commensal and pathogenic Gram-negative bacteria is the primary ligand that activates the IMD-NF-κB pathway, thereby triggering antimicrobial peptide (AMP) expression through pattern recognition receptors such as PGRP-LC and PGRP-LE [101]. However, to prevent chronic inflammation from continuous exposure to gut microbiota, *Drosophila* employs a layered network of negative regulatory mechanisms. Central to this are the amidase-type PGRPs, such as PGRP-LB, PGRP-SC1, and PGRP-SC2, which enzymatically degrade PGN fragments and thus reduce their immunogenic potential [98]. Deletion of these catalytic PGRPs results in hyperactivation of the IMD pathway and pathological immune responses even in the presence of innocuous commensal bacteria, highlighting their essential role in maintaining immune homeostasis and tolerance [98]. Complementing this enzymatic control, PGRP-LE functions as a regionalized sensor in the gut epithelium, recognizing monomeric PGN and orchestrating a balanced immune response through the induction of negative regulators such as PGRP-LB and Pirk, which attenuate Imd signaling and prevent systemic immune activation [92]. Loss of PGRP-LE disrupts this equilibrium, leading to overactivation of immune genes and tissue damage, thus underscoring its dual role in defense and tolerance [92]. At the transcriptional level, the homeobox gene Caudal acts as a molecular brake on the immune system by repressing NF-κB—dependent AMP gene expression in the posterior midgut, allowing the host to tolerate its commensal microbiota without triggering harmful inflammation [6]. Suppression of Caudal leads to ectopic AMP expression, disruption of the gut microbiota, epithelial apoptosis, and reduced host survival, thereby directly linking gene regulation to microbial community stability [6]. Thus, microbial PGN serves as a finely tuned immune cue in *Drosophila*, with pattern recognition, enzymatic degradation, feedback inhibition, and transcriptional repression operating in concert to achieve a delicate balance between antimicrobial vigilance and tolerance toward beneficial commensals.

Recent work shows that microRNAs (miRNAs) fine-tune *Drosophila* peptidoglycan-triggered immunity. One layer of control comes from Relish, the NF-κB transcription factor that not only drives antimicrobial peptide production but also stimulates miRNA expression. For example, Relish induces miR-317, which lowers levels of the receptor PGRP-LC, creating a negative feedback loop that limits Imd activation and helps restore immune balance [102]. Relish also induces miR-308, which targets the adaptor Tab2, reducing signaling strength in later infection stages and protecting flies from immune overdrive [103]. Other transcription factors also contribute to miRNA-mediated regulation. The growth regulator dMyc promotes miR-277, which suppresses both Imd and Tab2, two central signaling components. This down-regulation improves survival under infection by keeping immunity in check [104]. Some miRNAs enhance rather than repress immunity. The conserved miR-34 strengthens antibacterial defenses by silencing Dlg1 and Eip75B, while also linking immune activity to ecdysone, the major steroid hormone in flies. This places miR-34 at the crossroad of hormone signaling and immune regulation [105]. Genome-wide profiling adds to this picture. During *E. coli* infection, small RNA sequencing revealed many responsive miRNAs. Among them, miR-9a and miR-981 were shown to reduce antibacterial peptide production, again acting as brakes on the Imd pathway [106]. Thus, a network of miRNAs, including miR-317, miR-308, miR-277, miR-34, miR-9a, and miR-981, modulates different steps of the Imd pathway, from the PGN receptor to downstream adaptors and regulators. This layered regulation ensures that *Drosophila* mounts an effective defense against bacteria while avoiding immune overactivation.

#### Microbial Uracil and Lactate Promoting the Production of Reactive Oxygen Species (ROS)

**Figure 3:**
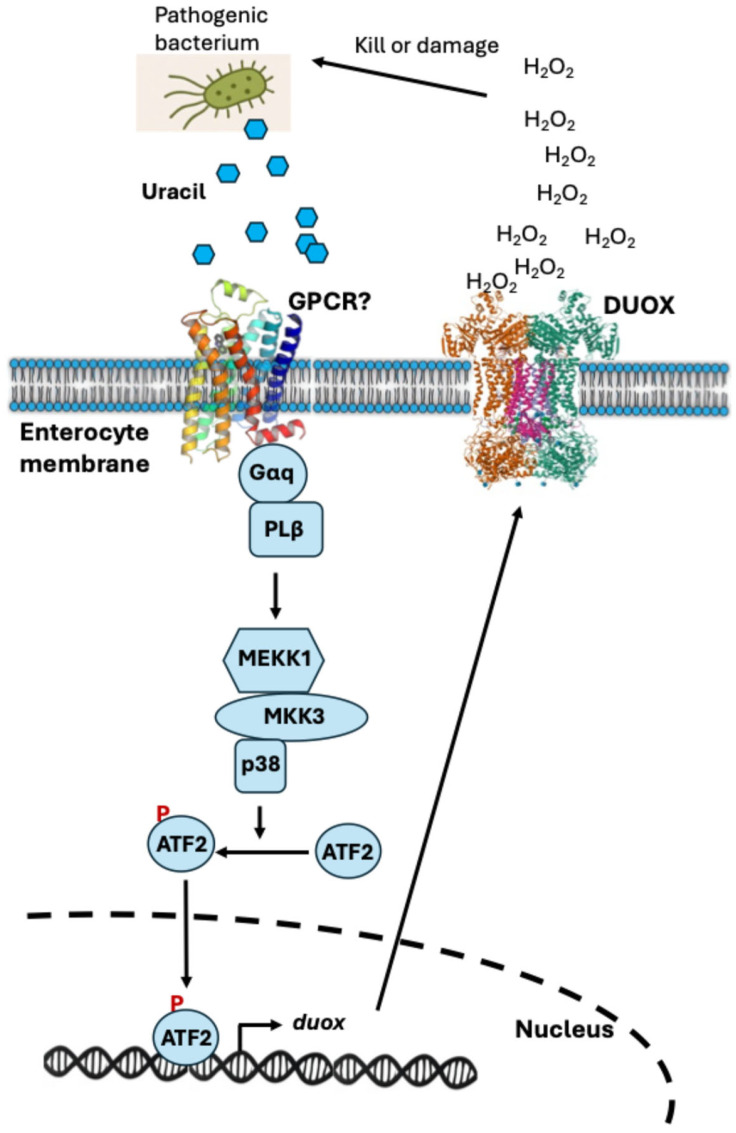
Uracil-Triggered Signaling Pathway for DUOX-Dependent Hydrogen Peroxide Production and Antimicrobial Defense in Enterocytes. Pathogenic bacteria release uracil, which is sensed at the enterocyte membrane, likely through a G-protein-coupled receptor (GPCR). This activates Gαq and PLβ, initiating a signaling cascade involving MEKK1, MKK3, and p38 kinase, leading to phosphorylation of the transcription factor ATF2. Phosphorylated ATF2 translocates to the nucleus to induce the expression of the *duox* gene. The DUOX protein is then produced and translocated to the membrane, where it generates hydrogen peroxide (H_2_O_2_). The locally produced H_2_O_2_ contributes to killing or damaging the pathogenic bacteria, mediating an antimicrobial response.

Uracil, a nitrogenous base released by certain pathogenic bacteria, plays a crucial role in *Drosophila* gut immunity. Unlike peptidoglycan, which is present in most bacteria (both beneficial and pathogenic), uracil secretion is primarily associated with pathogenic bacteria. The fly’s immune system recognizes uracil as a danger signal, allowing it to distinguish harmful microbes from harmless commensals. Upon detecting uracil in the gut lumen, likely through an as-yet-unidentified G protein-coupled receptor (GPCR), the enterocytes activate a signaling cascade involving Gαq, phospholipase Cβ (PLCβ), IP_3_, and calcium ions (Ca^2+^). This cascade stimulates the enzyme DUOX to produce ROS, especially hydrogen peroxide (H_2_O_2_), which acts rapidly to kill invading pathogens and limit infection [52,107] (Figure 3). This uracil-driven pathway not only provides an antimicrobial defense but also influences gut tissue repair by promoting intestinal stem cell proliferation. Flies with impaired DUOX activity exhibit reduced regenerative capacity and are more susceptible to gut infections, showing the importance of uracil sensing in maintaining gut integrity [107].

The specific receptor mediating uracil detection in *Drosophila* remains unidentified, representing an active area of research. There is a DUOX system in mammals, but the system does not sense microbial uracil [52]. However, parallels exist in mammalian nucleotide-sensing pathways like TLR8 [108]. Human TLR8 senses RNA degradation products in the of phagocytes’ endosome, and it can recognize viral and bacterial pathogens [108].

The enzyme NADPH oxidase (NOX) also contributes to ROS production in *Drosophila*, especially in response to lactate from beneficial commensal bacteria like *Lactobacillus*, helping to promote gut homeostasis and stimulate intestinal stem cell proliferation [109]. However, over-activation of NOX by overgrowth of *L. plantarum* resulted by PGRP-SD mutation causes intestinal damage, increased stem cell proliferation, and dysplasia, ultimately shortening lifespan [110].

### 4.3. Microbial Cyclic Dinucleotides (CDNs) Activating the STING Pathway

#### 4.3.1. Microbial CDNs in the Mammalian System

**Figure 4:**
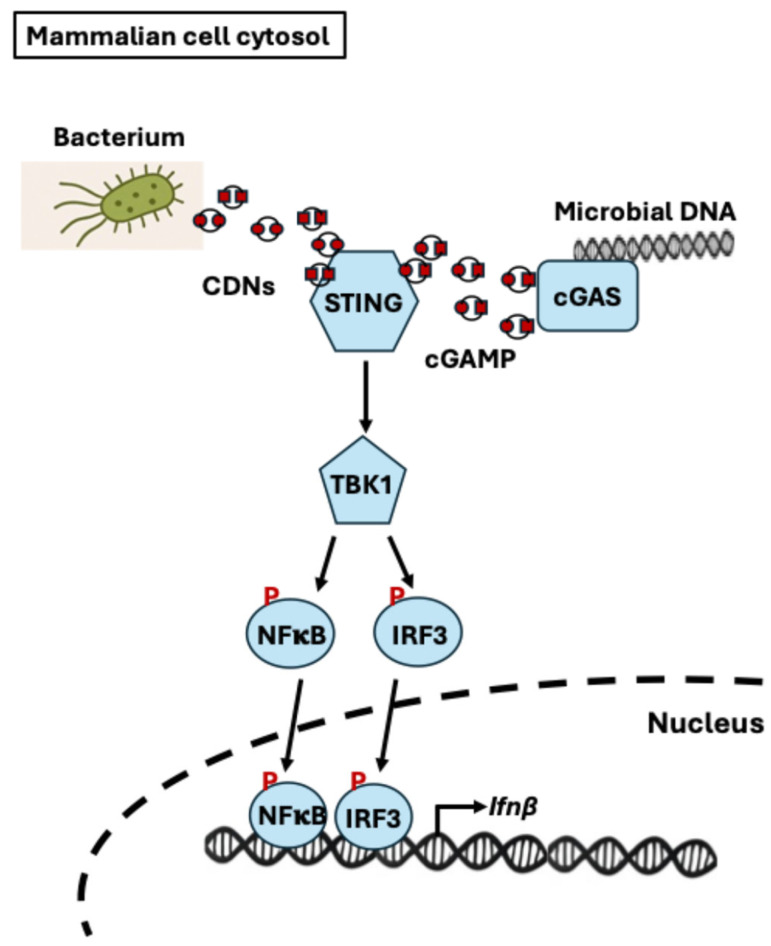
STING-Mediated Sensing of Bacterial Cyclic Dinucleotides (CDNs) and Microbial DNA in Human Cells. This diagram illustrates the STING (Stimulator of Interferon Genes) signaling pathway in the human cell cytosol in response to bacterial CDNs (c-di-AMP and c-di-GMP) and microbial DNA. Bacterial CDNs directly bind to STING, while microbial DNA is sensed by cyclic GMP-AMP synthase (cGAS), leading to the production of cyclic GMP-AMP (cGAMP), a second messenger that also activates STING. Upon activation, STING recruits and activates the kinase TBK1, which phosphorylates the transcription factors NF-κB and IRF3. These factors translocate into the nucleus, where they drive the transcription of type I interferon gene (*Ifnβ*), thereby initiating an innate immune response.

3′5′-cyclic di-adenosine monophosphate (c-di-AMP) and 3′5′-cyclic di-guanosine monophosphate (c-di-GMP) are bacterial signaling molecules known as second messengers that also function as microbe-associated molecular patterns (MAMPs) recognized by STING (Stimulator of Interferon Genes), the mammalian innate immunomodulatory system (Figure 4). STING also senses internally made 2’3’-cyclic GMP-AMP (cGAMP) produced by the cyclic GMP-AMP synthase enzyme (cGAS) activated by cytosolic bacterial or viral DNA. Although c-di-AMP and c-di-GMP are not typically secreted in large amounts, they can be released into the host environment during bacterial lysis or through active secretion, where they exert significant immunomodulatory effects [111,112,113,114].

c-di-AMP is produced by most Gram-positive bacteria, including human pathogens such as *Streptococcus pyogenes*, *Listeria monocytogenes*, and *Staphylococcus aureus*, and many Gram-negative bacteria [112,115,116,117]. When c-di-AMP enters mammalian cells, it binds to the cytosolic receptor STING, triggering a signaling cascade that activates TBK1 and IRF3. This leads to the production of type I interferons, such as IFN-β, which play a key role in antiviral and antibacterial defense. In addition to inducing type I interferons, in mouse macrophages, c-di-AMP has been shown to stimulate proinflammatory cytokines such as TNF-α and IL-6, and to induce chemokines including CCL3, CCL4, CXCL2, and CXCL10 [118]. Furthermore, in in vivo mouse models, c-di-AMP acts as a potent mucosal adjuvant by activating macrophages and dendritic cells via the STING pathway, leading to enhanced immune activation and recruitment [119].

c-di-GMP is primarily produced by Gram-negative bacteria, particularly those involved in biofilm formation, such as *Pseudomonas aeruginosa* and *Vibrio cholerae*, and also activates the STING pathway, leading to the production of type I interferons in mammals [113,114]. Studies in human and mouse models show that c-di-GMP drives dendritic cell maturation, upregulating surface molecules such as CD80, CD86, CD83, and MHC II, and induces proinflammatory cytokines including IL-12, IFN-γ, IL-6, IL-1β, and IL-23, thereby enhancing antigen presentation and stimulating robust Th1 and Th17 responses. These pathways are critical for defense against bacterial and fungal pathogens via STING-mediated signaling in dendritic cells (DCs) [120].

Commensal bacteria synthesize cyclic dinucleotides (c-di-AMP and c-di-GMP) at low levels as part of their normal physiology. For instance, intrinsic c-di-AMP was quantified via MS/MS in *Streptococcus mitis*, and lactic acid bacteria species are known to produce extracellular c-di-AMP capable of modulating host immune responses [121,122]. Genome-wide analyses reveal that canonical cyclic dinucleotide synthesis pathways, characterized by the DAC (di-adenylate cyclase, for the synthesis of c-di-AMP) and GGDEF-domain (for the synthesis of c-di-GMP) proteins, are widely conserved across both Gram-positive and Gram-negative bacteria, including common gut commensals in phyla like Firmicutes, Actinobacteria, Deltaproteobacteria, and *Escherichia coli* strains [123,124]. Although much of the CDNs research has focused on pathogenic bacteria, these emerging evidences suggest that during bacterial gut microbiota turnover or cell lysis, these molecules are released into the intestinal environment, where they can contribute to a baseline level of immune activation. Such low-level activation via pathways like STING ensures immune tolerance and prevents unnecessary inflammation. However, during infection or dysbiosis, increased bacterial turnover can lead to elevated CDN levels, which strongly activate the STING pathway, resulting in type I interferon production and recruitment of inflammatory leukocytes. While this heightened response is beneficial for pathogen clearance, excessive or chronic activation may contribute to tissue damage and promote inflammatory diseases such as inflammatory bowel disease (IBD). The STING pathway plays a dual role in colitis and colon cancer, exhibiting contrasting effects in each condition [125]. In colitis, activation of the STING pathway triggers innate immune responses through IRF3 and NF-κB, promoting inflammation and immune cell infiltration, which can exacerbate the disease. Consequently, inhibiting STING has shown promise in alleviating colitis symptoms. Conversely, during the transition to colon cancer, the expression of the STING pathway decreases, and its activation is associated with enhanced anti-tumor immune responses, such as increased infiltration of cytotoxic CD8+ T cells and reduced tumor invasiveness, correlating with better prognosis. In mammalian models, STING agonists derived from CDNs are being explored as vaccine adjuvants and cancer immunotherapies [126].

#### 4.3.2. Microbial CDNs in *Drosophila*

**Figure 5:**
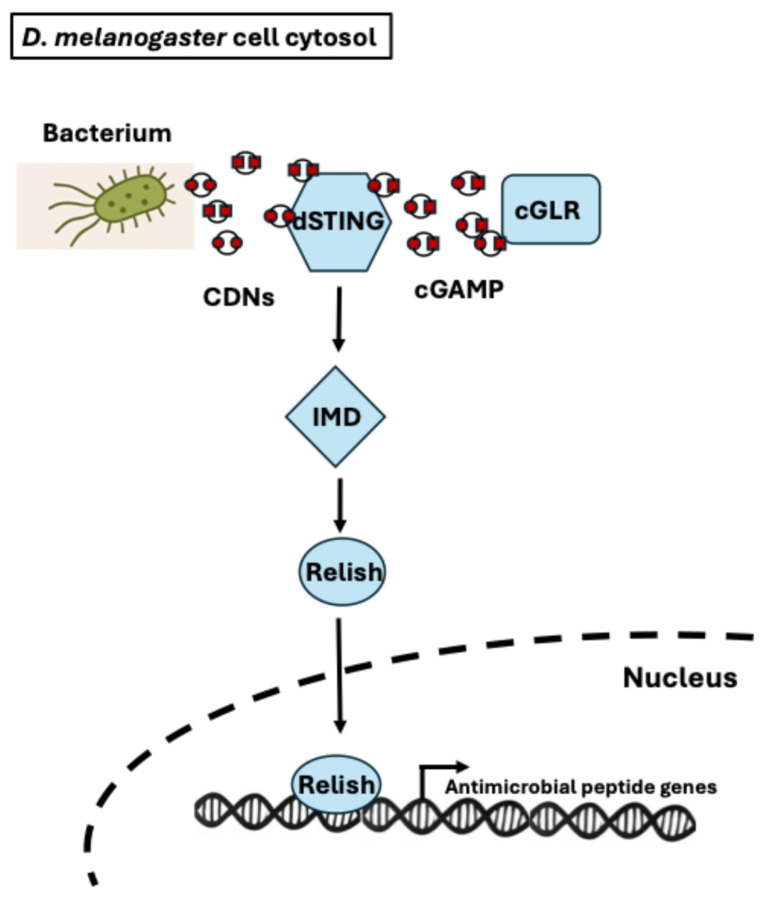
STING-Mediated Innate Immune Signaling Pathway in *D. melanogaster.* This schematic illustrates the dSTING signaling pathway in the *Drosophila melanogaster* cell cytosol in response to bacterial cyclic dinucleotides (CDNs). Bacterial CDNs (c-di-AMP and c-di-GMP) and cyclic GMP-AMP (cGAMP) produced by *Drosophila* cyclic GMP-AMP synthase-like receptors (cGLRs) activate dSTING. Upon activation, dSTING initiates signaling through the immune deficiency (Imd) pathway, leading to activation of the NF-κB transcription factor, Relish. Activated Relish translocates into the nucleus, where it induces the transcription of antimicrobial peptide genes, promoting an effective innate immune response.

Recent groundbreaking research in *Drosophila* has revealed that the fly’s immune system also detects CDNs produced during viral infections [127]. Specifically, *Drosophila* cGLRs (cyclic GMP-AMP synthase-like receptors), mammalian cGAS functional homologs, produce cyclic dinucleotides such as 2′3′-cGAMP and 3′2′-cGAMP, which activate the *Drosophila* STING (dSTING) to initiate antiviral immune responses [127]. Upon infection of *L. monocytogenes* that produces both c-di-AMP and c-di-GMP, dSTING activates the NF-κB transcription factor Relish, part of the Imd pathway, leading to a protective immune response that lowers both host mortality and bacterial burden [128]. This immune activation occurs in the absence of fly cGLRs. Even though there is no direct evidence that bacterial CDNs directly activate dSTING, this finding implies it and further suggests that STING is evolutionary conserved as a central antimicrobial defense mechanism in both flies and mammals. Interestingly, a novel cyclic dinucleotide called 2′3′-c-di-GMP was identified as a potent STING agonist in *Drosophila*, highlighting the diversity of these signaling molecules [127]. The role of bacterial CDNs in *Drosophila* immunity may reveal new insights into how invertebrate immune systems detect and respond to bacterial infections (Figure 5). A recent study using *Drosophila* demonstrates that the gut resistance to infection is significantly influenced by signals originating from the commensal flora [129]. In the study, removal of these microbes makes flies more susceptible to oral viral infections, and administering bacterial CDNs to flies with depleted microbiota restores their increased susceptibility, indicating that microbial CDNs can activate protective immune pathways. This immune enhancement relies on the presence of dSTING and dTBK1, ultimately driving NF-κB-dependent gene activation. Notably, it was identified that the apical nucleoside transporter CNT2 as critical for this oral CDN-mediated protection. These results show a key role for bacterial CDNs in conditioning gut immune responses.

## 5. The Impact of Gut Microbiota on *D. melanogaster*

### 5.1. Metabolism, Nutritional Homeostasis, Growth, and Development

The gut microbiota of *D. melanogaster* strongly affects host metabolism, nutrition, and growth. Under nutrient-poor conditions, *L. plantarum* accelerates larval growth by enhancing amino acid assimilation and activating the TOR signaling pathway, which governs nutrient-dependent growth [41]. In return, larvae promote bacterial survival by releasing compounds such as N-acetylglucosamine, which help *L. plantarum* withstand the stressful gut environment. Through a cycle of ingestion, release, and reingestion, the bacteria persist in the food substrate and repeatedly colonize larvae. This reciprocal interaction ensures improved larval growth on poor diets and stable bacterial populations, establishing a facultative nutritional mutualism [130]. *A. pomorum* influences development, body size, and metabolism by producing acetic acid that alters insulin/IGF signaling [131].

Parallels to these mechanisms exist in mammalian systems. In humans and rodents, short-chain fatty acids (SCFAs) such as acetate, produced by gut commensals including *Bacteroides*, *Lactobacillus*, and *Faecalibacterium*, act as key signaling molecules via G-protein-coupled receptors linking the microbiota to host metabolism [132,133]. SCFAs engage enteroendocrine cells (EECs) in the intestine via receptors such as GPR41 (FFAR3) and GPR43 (FFAR2), triggering the release of metabolic hormones such as PYY and GLP-1, which influence satiety (through PYY) and insulin secretion (through GLP-1) [133,134]. SCFAs also activate AMPK and PPAR-γ, key regulators of insulin sensitivity and energy homeostasis [133]. More broadly, microbiota–EEC crosstalk represents a conserved endocrine conduit in mammals, where gut-derived signals modulate glycemia, lipid metabolism, and appetite regulation [134].

Thus, across species, a shared biological process emerges. Microbial metabolites such as SCFAs function as interkingdom messengers that connect the gut microbiome with the host’s endocrine and nutrient-sensing networks [133]. In *Drosophila*, acetate produced by *A. pomorum* activates IIS, promoting development and metabolic balance [44]. In mammals, SCFAs, including acetate, stimulate EECs to regulate GLP-1 release and insulin activity [134]. Both systems employ a conserved strategy in which microbial carbon flux influences hormone-based metabolic regulation. Moreover, nutrient-sensing growth control is maintained through analogous pathways: commensals in flies enhance TOR-dependent growth [41], and mammalian microbiota modulate insulin and mTOR signaling through SCFA-mediated nutrient sensing [133].

Gut microbes also provide missing nutrients—*Acetobacter* species supply thiamine and, together with *L. plantarum*, adjust amino acid and vitamin needs to make up for dietary gaps [131,135]. Commensal bacteria can also change the nutritional quality of food by increasing protein relative to carbohydrates, improving protein quality, and boosting tryptophan, all of which support development and lifespan under nutrient restriction [136,137]. They also protect against metabolic stress by saving B vitamins, preventing excess fat buildup on high-sugar diets, and shifting energy use differently in males and females [2,138]. Microbial abundance also matters, as higher amounts of bacteria can rescue development and extend lifespan on protein-poor diets [137]. Overall, the *Drosophila* gut microbiota helps balance nutrition, provides missing nutrients, and works with host signaling pathways to ensure proper growth and development under changing diets.

### 5.2. Behavior and Neural Function

The gut microbiota of *D. melanogaster* plays an active role in shaping its feeding behavior through a combination of metabolic, sensory, and behavioral pathways. Far from being passive symbionts, bacterial communities in the fly gut influence what, when, and how flies choose to eat. One major mechanism involves the modulation of olfactory-driven food selection. Both larval and adult flies exhibit attraction toward food sources containing their native bacterial symbionts, particularly *A. pomorum* and *L. plantarum* [139]. When the gut microbiota was experimentally removed, these preferences disappeared, indicating that microbial composition determines the chemosensory cues guiding food-seeking behavior. In addition, flies deprived of their microbiota showed reduced discrimination between food patches seeded with different bacteria, suggesting that gut microbes calibrate the host’s sensory systems to recognize beneficial microbial odors. Another layer of regulation arises from the interaction between gut microbes and dietary amino acid balance [42]. When flies are deprived of essential amino acids (EAAs), they normally exhibit a strong appetite for protein-rich foods such as yeast. However, when *L. plantarum* and *A. pomorum* were present, this compensatory feeding drive was suppressed. These commensal bacteria appear to buffer the host against nutrient imbalance, maintaining feeding stability and supporting reproductive success. Expanding on this finding, it was uncovered that a metabolic cross-feeding relationship between *A. pomorum* and *L. plantarum* that further explains their behavioral influence [140]. The two species exchange metabolites, *L. plantarum* produces lactate, which *A. pomorum* converts into amino acids, enabling them to thrive even when dietary amino acids are scarce. The metabolic cooperation between these microbes sustains a stable gut community and generates metabolites that alter the host’s feeding preferences, reducing protein cravings and improving reproductive output. Studies have also expanded the microbial scope beyond bacteria. Yeasts within the fly gut and environment can affect feeding decisions [74]. Flies tend to favor naturally associated yeast species over *S. cerevisiae*, suggesting that ecological and microbial familiarity influences food choice. This further emphasizes that *Drosophila* feeding behavior emerges from complex interactions between multiple microbial taxa rather than single-species effects.

Beyond nutrient-related feeding control, diet-induced changes in gut microbiota can also reprogram behavioral preferences; dietary differences (e.g., starch- vs. sucrose-based diets) rapidly alter the composition of the *Drosophila* microbiome, leading to distinct behavioral outcomes [141]. Flies maintained on starch media developed a preference for mates with similar microbial profiles, a change mediated by the proliferation of *L. plantarum*, implying that diet-driven microbial shifts can modify both food-related and social behaviors.

The gut microbiome of *D. melanogaster* profoundly influences both aggression and locomotor activity, acting through the octopaminergic system, which serves a role similar to noradrenaline in mammals. In a study by Jia et al., aggressive behaviors of germ-free flies were markedly reduced, with males showing fewer lunges and longer latency before attacking [142]. This behavioral dampening coincides with a significant decrease in octopamine levels and reduced expression of tyrosine decarboxylase 2 (Tdc2), an enzyme essential for octopamine synthesis. Reintroducing commensal bacteria, particularly *L. plantarum*, restores normal aggression, but only if the microbes are metabolically active. The effect depends on early developmental colonization (48–96 h post-egg laying), suggesting that microbial cues shape neurodevelopmental circuits during a critical period. Artificially boosting octopaminergic neuron activity or supplying octopamine chemically fully rescues the aggression deficit, confirming that the microbiome enhances octopaminergic signaling to promote assertive behavior. In contrast, Schretter et al. found that the absence of gut microbes produces hyperactivity; flies move faster, restless, and display longer walking bouts [21]. This overactivity can be normalized by *L. brevis*, whose secreted enzyme xylose isomerase corrects locomotion by altering host sugar metabolism, especially trehalose levels. Xylose isomerase and *L. brevis* reduce expression of Tdc2 and Tβh, suppressing octopamine production. Artificial activation of octopaminergic neurons or administration of octopamine negates the bacterial effect, indicating that *L. brevis* reduces locomotion by decreasing octopaminergic activity.

### 5.3. Immunity

The gut microbiota of *D. melanogaster* has a major influence on how the immune system works, both inside the gut and throughout the body. Bacteria in the intestine constantly release small molecules like peptidoglycan, which are detected by host receptors such as PGRP-LC and PGRP-LE. This activates the Imd pathway and leads to the production of antimicrobial peptides (AMPs) that kill harmful microbes. The Toll pathway acts in a similar way but is mainly triggered by Gram-positive bacteria and fungi [92,143]. Because beneficial microbes also produce these signals, the fly has mechanisms to avoid attacking its own microbiome. For example, enzymes like PGRP-LB and PGRP-SC break down excess peptidoglycan, and the transcription factor Caudal helps reduce AMP expression in the gut, preventing unnecessary inflammation against beneficial bacteria [92]. Another important defense is the DUOX system, which makes reactive oxygen species (ROS). These molecules can quickly destroy invading bacteria and also signal for gut tissue repair, but if too much ROS is produced it can damage the intestine [52]. Gut microbes also influence the gut’s hormone-producing enteroendocrine cells. These cells act as a link between microbial sensing, metabolism, and barrier function, helping the host balance energy use with immune protection [31]. In addition, antiviral defenses in the gut depend on nutrient-sensitive ERK signaling, which is triggered by microbial and dietary cues. This pathway helps block viruses from spreading in the intestinal epithelium [144]. In short, the *Drosophila* gut microbiota “trains” the immune system by providing constant signals that must be carefully managed. The fly uses tolerance mechanisms to protect helpful microbes while still keeping powerful antibacterial and antiviral defenses ready. This balance allows the gut to remain healthy and at the same time contributes to whole-body homeostasis [143,145].

### 5.4. Tumorigenesis

Research in *D. melanogaster* shows that the gut microbiota is not passive but actively influences tumor development. In models with impaired Notch signaling, tumor formation is markedly reduced under germ-free conditions, demonstrating that microbial communities are required for efficient tumor initiation and progression [53]. Different bacterial species exert distinct effects. For instance, *L. brevis* strongly promotes tumor growth by driving intestinal stem cell proliferation, altering integrin distribution, and favoring symmetric stem cell divisions, which expand the pool of undifferentiated cells. These changes accelerate the formation of large, multilayered tumors. In contrast, its close relative *L. plantarum* does not trigger similar tumor-promoting activity, pointing to species-specific roles among commensals [53]. Tumorigenesis also destabilizes host–microbe interactions. Barrier breakdown in the intestine, such as that caused by defective Bone Morphogenetic Protein (BMP) signaling, results in higher bacterial loads, reduced microbial diversity, and overrepresentation of *Acetobacter* species. This dysbiotic state further stimulates regenerative signaling and fuels tumor progression. Suppression of JNK signaling or removal of microbes can restore barrier integrity and limit tumor expansion, highlighting the feedback loop between microbial imbalance and cancer growth [15].

## 6. Conclusions

The gut microbiota of *D. melanogaster* provides a simplified yet powerful system for uncovering fundamental principles of host–microbe interactions. Despite its low diversity, dominated mainly by *Lactobacillus* and *Acetobacter* species, this community profoundly influences host physiology. Microbes modulate nutrient acquisition, regulate insulin/TOR signaling, and buffer against dietary imbalances, thereby supporting growth and metabolic homeostasis. At the same time, microbial molecules such as peptidoglycan, acetate, uracil, and cyclic dinucleotides engage conserved immune pathways, including Imd, Toll, DUOX, and STING, highlighting the dynamic balance between immune activation and tolerance that preserves tissue integrity and systemic health.

Beyond metabolism and immunity, the microbiota shapes behavior, reproduction, and even susceptibility to diseases such as cancer, revealing its broad physiological influence. Dysbiosis or inappropriate immune responses disrupt this balance, accelerating aging, promoting chronic inflammation, and driving tumorigenesis.

Studies in *Drosophila* highlight the microbiota’s role as both a nutrient partner and an immune modulator, illustrating conserved strategies that extend to mammalian systems. By leveraging its genetic tractability and ecological relevance, *Drosophila* continues to serve as a key model for disentangling the complex, bidirectional relationships between gut microbes and host biology, with implications for understanding human health and disease.

## Figures and Tables

**Table 1:** Key Peptidoglycan Recognition Proteins (PGRPs) in *Drosophila* Gut Immunity.

PGRP Receptor	Type	Recognized PGN	Associated Microorganisms	Immune Pathway Activated	Immune Modulation Mechanism	Ref.
PGRP-LC, Three isoforms (LCa, LCx, and LCy) ^a^	Transmembrane receptors.	DAP-type PGN	Gram-negative bacteria and some Gram-positive bacteria such as *Bacillus*	Activate Imd → Relish → AMP production	Detects pathogens and commensals; initiates activation; maintains tolerance in dysbiosis; regulated by receptor degradation (rPGRP-LC) ^b^	[88,89,90]
PGRP-LE	Secreted/Cytosolic	DAP-type PGN	Gram-negative bacteria, and intracellular pathogens like *Listeria*	Downregulate Imd/Rel pathway	Works synergistically with PGRP-LC; down-regulated by amidases such as PGRP-LB in dysbiosis (negative feedback regulation)	[88,90,91,92,93]
PGRP-SA	Secreted	Lys-type PGN	Gram-positive bacteria	Activate Toll → Dif → AMP production	Systemic immune response; amplifies responses to gut-derived signals; L,D-carboxypeptidase activity for diaminopimelic acid-type tetrapeptide PG fragments but not lysine-type PG fragments	[94,95]
PGRP-SD	Secreted	DAP-type PGN	Gram-negative bacteria and some Gram-positive bacteria such as *Bacillus*	Upregulate Imd/Rel pathway	Acts upstream of PGRP-LC as an extracellular receptor to enhance peptidoglycan-mediated activation of Imd signaling by enhancing the localization of peptidoglycans to the cell surface. Antagonizes the action of PGRP-LB to fine-tune the intensity of the immune response.	[99]
PGRP-LB	Secreted Amidase removing peptides from PGN.	Both PGN types	Most bacteria, including commensals and pathogens	Downregulate Imd/Rel pathway	Degrades PGN fragments; limits immune overactivation; maintains homeostasis	[91,92,98]
PGRP-SC2	Secreted Amidase	DAP-type PGN	Gram-negative bacteria and some Gram-positive bacteria such as *Bacillus*	Negative regulator of Imd/Rel pathway	Degrades PGN fragments; prevents dysbiosis, promotes tissue homeostasis, and extends lifespan.	[100]

^a^ Each isomer consists of the same intracellular domain linked to a different extracellular domain. ^b^ Alternative splice variant of PGRP-LC. It induces endosomal degradation of activating and regulatory PGRP-LC receptors.

## Data Availability

The original contributions presented in this study are included in the article. Further inquiries can be directed to the corresponding author.
